# Clonal Plants as Meta-Holobionts

**DOI:** 10.1128/mSystems.00213-18

**Published:** 2019-03-19

**Authors:** Nathan Vannier, Cendrine Mony, Anne-Kristel Bittebiere, Kevin R. Theis, Eugene Rosenberg, Philippe Vandenkoornhuyse

**Affiliations:** a Université de Rennes, CNRS, UMR6553 EcoBio, Rennes, France; b Université de Lyon 1, CNRS, Villeurbanne, France; c Department of Biochemistry, Microbiology and Immunology, Wayne State University, Detroit, Michigan, USA; d Department of Molecular Microbiology and Biotechnology, Tel-Aviv University, Tel Aviv, Israel; Cornell University

**Keywords:** holobiont, plant-microbe interactions, symbiosis

## Abstract

The holobiont concept defines a given organism and its associated symbionts as a potential level of selection over evolutionary time. In clonal plants, recent experiments demonstrated vertical transmission of part of the microbiota from one ramet (i.e., potentially autonomous individual) to another within the clonal network (i.e., connections by modified stems present in ∼35% of all plants). Because of this heritability, and potentially reciprocal exchange of microbes between generations of ramets, we propose to extend the existing holobiont framework to the concept of meta-holobiont. A meta-holobiont is a network of holobionts that can exchange biomolecules and microbiota across generations, thus impacting the fitness of both biological scales: holobionts and meta-holobionts. Specifically, meta-holobiont dynamics can result in sharing, specialization, and division of labor across plant clonal generations. This paper, which coins the meta-holobiont concept, is expected to stimulate discussion and to be applied beyond the context of networked clonal plants (e.g., to social insects).

## Abstract (precis)

The holobiont concept defines a given organism and its associated symbionts as a potential level of selection over evolutionary time. In clonal plants, recent experiments demonstrated vertical transmission of part of the microbiota from one ramet (i.e., potentially autonomous individual) to another within the clonal network (i.e., connections by modified stems present in ∼35% of all plants).

## OPINION/HYPOTHESIS

## THE GENOME IS NOT THE ONLY GENETIC SOURCE OF PHENOTYPIC VARIATION AMONG MACROORGANISMS

The theoretical basis of neo-Darwinian evolution (i.e., the modern synthesis) is that a genetic variant, such as a mutation, is a stochastic event having neutral, advantageous, or disadvantageous consequences for fitness and that via natural selection, advantageous variants can increase in relative frequency within large populations (1). This sorting of genetic variants by natural selection operates through individual phenotypes. It is thus generally believed that an organism, its functions, and its ability to adjust to environmental constraints can be addressed through analysis of its genome (i.e., the information repository of the organism). Behind this idea is the assumption that the phenotypes of organisms are, in large part, programmed through gene expression. This view of genomes is an oversimplification (2). For instance, there are examples of physical transience (i.e., genome composition and stability do not remain fixed at all times in every organism), as in ciliates (e.g., *Oxytricha*) (3). Less anecdotally, there is additional modifying information regarding phenotype expression. First, epigenetic markers (i.e., DNA methylation, histone modification, histone variants, and small RNAs) can be passed from one generation to another and are reversible (4). These markers induce a suite of interacting molecular mechanisms which impact gene expression and function and thus phenotypes, without changing the DNA sequence (5). Second, all macroorganisms (i.e., animals and plants) interact with symbiotic microbes throughout most of their life stages. Such associations have a deep impact on phenotypic variation and directly affect host fitness (6, 7) (see Fig. S1 in the supplemental material). However, a large proportion of population genetics studies, including in plants, only consider the host genome despite the microbe-free plant being “an exotic exception rather than the biologically relevant rule” (8). Thus, plant phenotypic variations are often, and likely erroneously, attributed to host genome variants alone, and the neo-Darwinian idea of host species often views symbiotic microbes as an environmental factor rather than a genetic one. Neo-Darwinism remains valid because the environment is often a stronger contributor to microbiome structure than is host genotype. However, this is not always so, and the ubiquity and impact of the microbiome in host biology challenge the current concept of self (9) and call for a holistic approach for furthering understanding of host-microbe associations.

10.1128/mSystems.00213-18.2 FIG S1 Glechoma hederacea phenotypic plasticity induced by its microbiota composition. All the plants have the same genotype (i.e., they are from the same clone), are growing under controlled conditions (light, temperature, hygrometry, and water supply), and differ only in a single component of their microbiota (i.e., mycorrhizal colonizer). The picture shows a clone of G. hederacea that has been inoculated with an isolate of Rhizophagus invermaius, Glomus diaphanum, or Archaeospora trappei (R.in, G, or A, respectively). T is the nonmycorrhized control. Download FIG S1, TIF file, 1.3 MB. Copyright © 2019 Vannier et al. 2019 Vannier et al. This content is distributed under the terms of the Creative Commons Attribution 4.0 International license.

## HOLOBIONT AND HOLOGENOME CONCEPTS

A given macroorganism can no longer be considered an autonomous entity but rather as the product of host-microbe associations forming a holobiont (10), with their collective genomes comprising the hologenome. The holobiont encompasses not only the host and its obligate symbionts but also its facultative symbionts and the emergent properties arising from these multivarious and dynamic associations (11). This implies that genetic variations arising in the host or any microbial genome within the holobiont are potential sources of hologenomic variation that can be phenotypically neutral, deleterious, or beneficial for the holobiont (10, 12). This is the keystone of the hologenome concept of evolution (12, 13). Genetic changes can also be introduced to the hologenome, during the lifetime of the host, through immigration and emigration of microbial symbionts. For example, a plant can recruit a microorganism(s) within the phyllosphere and rhizosphere to buffer environmental constraints (14). A plant’s control over its microbiota includes the secretion of exudates within the rhizosphere as well as immunity within the endosphere (14). These elements promote the capacity for plants to not only negotiate recurrent interactions with their beneficial and cooperative microbial partners but also to mitigate the influx of potential pathogens into the plant holobiont. Modifications of the microbiota in a plant holobiont could alter the hologenome and rapidly lead to the acquisition of new functions enabling adjustment to fluctuating biotic and abiotic environmental stressors. In natural ecosystems, for most plants, seed dispersion occurs over short distances (15), and seedlings are likely to come into contact with the same populations of microorganisms as their parent plant (16). Recruited microorganisms can be vertically (17, 18) or pseudovertically transmitted (i.e., acquired by offspring from the same environmental populations as the parents) (14). Coupled with vertical transmission in particular, the change of microbiota of individuals during their lifetime, in ways that promote plant adjustment to the environment (7), could translate to Lamarckian deterministic evolution (i.e., inheritance of acquired characteristics) (19). In this context, the assembly of the microbiota constitutes a large portion of the repository of genetic information transmitted between generations.

Holobionts and hologenomes are becoming increasingly recognized as biological units (20). The current debate surrounding holobionts and hologenomes is not whether they exist but rather whether they are consistently units of selection in evolution (21). Although some of the debate has been a product of semantic differences (11), criticisms of the hologenome concept of evolution are that the hypothesis is host centric (i.e., more useful for studying the evolution of hosts than of their microbial populations), that vertical transmission and thus the heritability of the microbiota is rare, and that the selective interests among microbial symbionts and between symbionts and their hosts are likely to be too discordant for selection to act at the level of the hologenome (21, 22). The purpose of this paper is not to debate the validity of the hologenome as an evolutionary unit but rather to develop a new perspective for networked clonal organisms within the holobiont framework.

## HOLOBIONT MICROBIOTA ASSEMBLY

Natural selection can act on the holobiont phenotype and, to the extent that its microbial members are heritable or reassembled with fidelity across host generations, shape the microbiota assembly of the holobiont. Notably, however, the heterogeneity in microbiota community structure observed among hosts can also be due to neutral processes. Ecological theories predict that both niche partitioning (through environmental filtering or species interaction sorting [23]) and neutral processes (24) drive community assembly and thus explain the observed patterns in community diversity. The neutral and stochastic versus the deterministic nature of host-microbiota assembly has been debated (25). In a random community assembly model (25), a large proportion of the microbiota is recruited directly from the environment without any filtering or interactive sorting. However, because the functions performed by the microbiota can be important for holobiont fitness, specialization at the metabolic level of the microbiome would be expected, and there should be selective pressure on the hologenome. Thus, a deterministic model of host microbiota assembly is likely to be observed (i.e., non-random assembly [25]). Deterministic assembly can come about through two mechanisms: first, recruitment, filtering, and policing by the host plant; second, for particular microorganisms within the microbiota, the relaxation of selection pressure on “useless” genes and the accumulation of mutations in these particular genes leading to a loss of functions.

## PLANT HOLOBIONTS AND HOLOGENOMES: A SPECIAL CASE

Plants are sessile macroorganisms. This conditions their interactions with their environment. First, they are unable to escape environmental constraints and need to adjust to abiotic (e.g., resource limitation) and biotic (e.g., competition and predation) stressors. Second, plants, like other sessile organisms, influence their own environmental conditions. For instance, they deplete the mineral and water resources of their habitat and modify the microclimate and local soil characteristics through the development of above and belowground organs (26). This retroaction of plants on their environment drives local environmental fluctuations over seasonal and annual time scales. In the two above-described situations, the recruitment of microorganisms within the microbiota could enable plants to acquire new ecological functions, such as increasing resource acquisition or buffering against fluctuating environmental conditions (27). Changes in the microbiota composition across seasons or years may thus reflect, in addition to changing ambient conditions, the temporal needs of the plants. Considering that environmental changes can be either continuous or disruptive and occur over short or long time scales, the recruitment of microorganisms represents opportunities for quick and long-term adjustments and adaptations to environmental constraints that are less costly for the plant than plastic responses (28). Plant-associated microorganisms can thus condition plant survival and fitness (7, 14). However, it must be noted that trade-offs would be inherent in the recruitment of microorganisms. Specifically, unless there are established mechanisms in place to limit recruitment and colonization to those microorganisms conferring phenotypic benefit to the holobiont, then colonization by parasites and pathogens could also occur. It is therefore expected that such sentry mechanisms would be under strong selective pressure among clonal plant holobiont populations.

**FIG 1:**
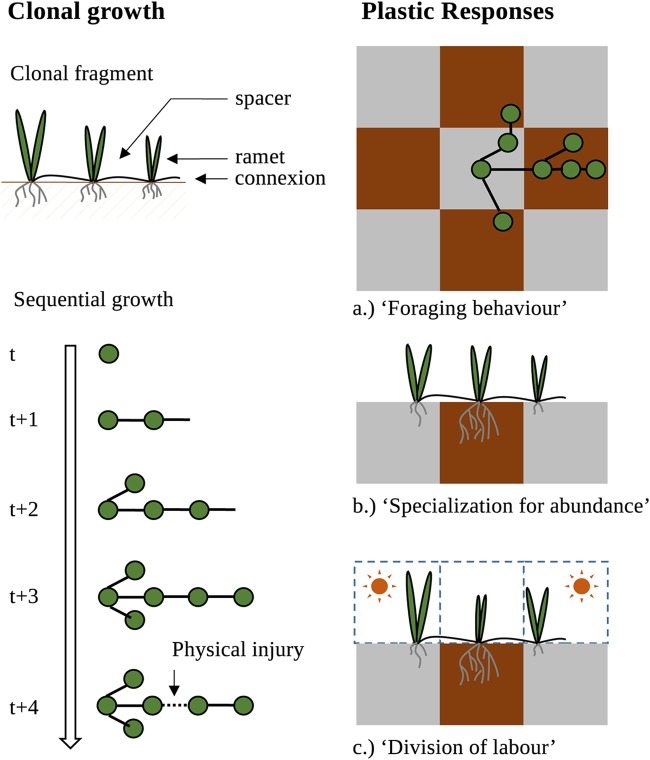
Organization of a clonal plant, sequential growth, and plastic responses in heterogeneous environments. (Top left) Clonal fragments are composed of ramets linked through connections that can be aboveground or belowground (see supplemental material for vocabulary definitions). (Bottom left) Clonal growth is sequential and multiplies ramets and connections. A clonal fragment can be split due to physical injury or connection life span. (Right) Clonal fragments display particular plastic responses unique to clonal plants that are “foraging behavior” (a), “specialization for abundance” (b), and “division of labor” (c). Foraging behavior consists of aggregating the ramets in rich patches (brown) and avoiding poor patches (gray). Plastic responses may include connection length, direction of growth, and intensity of ramification. Specialization for abundance describes when ramets locally invest in organs that uptake the most abundant resource and share this resource to the other ramets thanks to physiological integration. Division of labor is an extension of specialization when the environment presents two resources that are negatively correlated (such as light and soil resources). Ramets thus display high aboveground allocation under high-light and low-nutrient conditions, whereas the belowground allocation is high under the reverse conditions.

Plants are modular organisms; they can be considered discrete units of organization (modules) forming a network system (29). Their growth is iterative through undifferentiated and totipotent tissues (meristems) (Fig. 1). In plants, modularity is expressed at different levels of integration ranging from simple subunits such as a leaf, root, or flower to more complex units such as ramets (i.e., potentially autonomous clonal units composed of roots and shoots modules) produced by clonal multiplication (see supplemental material). In temperate ecosystems, approximately 70% of plants are clonal (29). A common form of clonality (present in ∼50% of clonal plants or ∼35% of all plants) involves growth as a reticulated network linking clonal plant holobionts via horizontal modified stems that develop aboveground (i.e., stolons) or belowground (i.e., rhizomes) (30) (Fig. 1). Such reticulated networking (i.e., clonal fragment *sensu* [30]) allows an individual to generate a number of genetically identical ramets that correspond ultimately to separated physical and physiological entities if connections are damaged or senesced (31).

Environmental sampling and information sharing contribute to adjust the network structure and ramet growth to local stresses and environmental heterogeneity (29), thereby impacting the fitness of the clonal fragment. In response to patchy distribution of favorable habitats, plastic changes have been reported in (i) the architecture of the network and (ii) the morphology of the ramets (Fig. 1). When resource distribution is patchy, clonal individuals aggregate ramets by internode shortening and increased branching in resource-rich patches and avoid resource-deficient patches through spacer elongation (i.e., foraging behaviors) (Fig. 1) (32). In parallel, in response to a patchy distribution of resources, ramets can specialize in the acquisition of the most abundant resource and share it within the network for the benefit of the entire network (“specialization” behavior) (Fig. 1) (33). This specialization process can lead to the “division of labor” (33), which occurs when the spatial distributions of two resources are negatively correlated (Fig. 1) (33). These two types of plasticity are specific to clonal plant networks.

**FIG 2:**
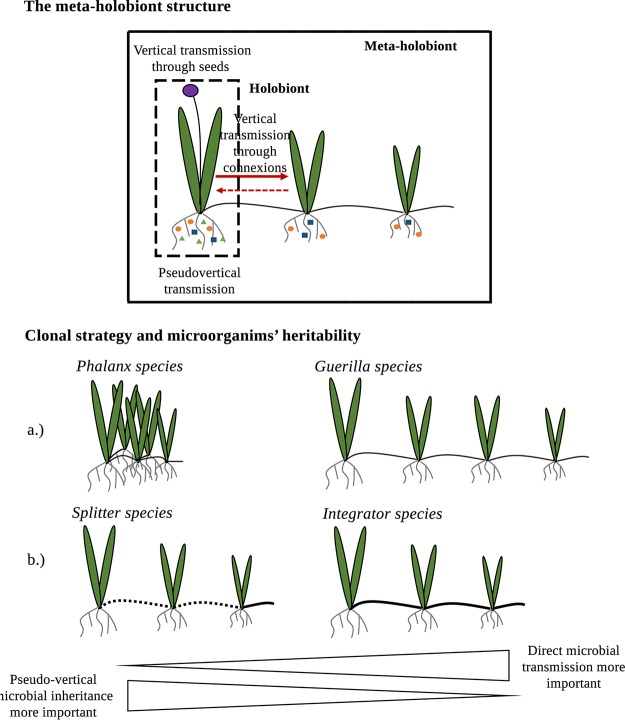
Meta-holobiont and importance of transmission between holobionts. (Top) The structure of the meta-holobiont is explained. Colored dots, triangles, and squares correspond to epiphytic or endophytic microorganisms forming the root microbiota. Note that all plant compartments (i.e., roots and shoots) are colonized by microorganisms even if not illustrated in this figure. Transmission can be vertical through seeds (1) or through connections (2) and pseudovertical (3). (Bottom) The relative importance of 2 and 3 depending on the clonal strategy in terms of architecture of the clonal network (phalanx versus guerrilla) (a) and life span of the connection (splitter versus integrator) (b).

Ramet sampling may include more than sampling of only the resources in the environment. The clonal network may also allow the host plant to sample microorganisms within the soil. This process has never been taken into account to understand the microbiota assembly in clonal plants, despite the recognized importance of microbiota for plant fitness. Pseudovertical and vertical transmissions of microbiota would likely ensure the presence of suitable symbionts and thus limit foraging costs associated with clonal exploration for plants (16). The question of microbiota transmission is therefore of fundamental importance. Transgenerational reassembly of microorganisms associated with ramets may depend on the physical scale between them. In phalanx networks (Fig. 2 and supplemental material for definition), clonal offspring are expected to be in contact with a similar pool of microorganisms as the mother plant (i.e., strong pseudovertical transmission). Conversely, in guerilla networks (see supplemental material), weak pseudovertical transmission is expected (i.e., offspring encounter microorganisms that differ from those of the parents) (Fig. 2). However, it has recently been demonstrated that clonal plants are able to transmit a core microbiota (i.e., a fraction of the mother’s microbiota) containing *Bacteria* and fungi, but not *Archaea*, to progeny through their stolons (34, 35) (Fig. 2). Consistent with the hologenome concept, this vertical transmission of a subset of the mother’s microbiota could provide an insurance of habitat quality for the offspring and serve as a viable route for the inheritance of acquired characteristics (and genetic material).

## BEYOND HOLOBIONTS: CLONAL PLANTS AS META-HOLOBIONTS

Considering that a particular clonal ramet is colonized by a complex microbiota, that at least part of the microbiota is transmitted between clonal generations, and that these transmitted microorganisms (e.g., arbuscular mycorrhizal [AM] fungi) (35) can alter the fitness of the ramet, clonal plants satisfy the tenets of the hologenome concept (12, 13). However, the plant clonal network represents an additional level of organization in which holobionts (in this case, ramets) and hologenomes are interconnected and integrated in a higher modularity model (i.e., the networked clonal fragment). To study this level of organization, we introduce the concept of meta-holobiont (Fig. 2).

## THE META-HOLOBIONT CONCEPT

Bordenstein and Theis (10) proposed a framework of ten principles for holobionts and the hologenome theory which do not change the rules of evolutionary biology but redefine what a macroorganism is. The meta-holobiont concept is primarily dedicated to understanding and defining the unique aspect of network-forming clonal macroorganisms (i.e., like reticulated clonal plants) through which the individual holobionts can share biomolecules (31) and microorganisms (34, 35), potentially across generations in a reciprocal manner (Fig. 2). The meta-holobiont concept posits that passive or active transfers of microorganisms, resources, and information between holobionts through a network can affect the fitness of individual holobionts as well as meta-holobionts more broadly. The meta-holobiont concept also posits the existence of specific physical host structures for constructing the network and thereby facilitating these transgenerational exchanges of closed microbial populations among holobionts that are genetically identical at the level of host genome. The physical structures linking clonal plant holobionts are of critical importance as they serve as support for the plastic responses developed at both holobiont and meta-holobiont scales. This physical and reliably structured conduit for repeated and integrated exchanges of microorganisms and their products among genetically similar ramets is what differentiates multiple generations of holobionts from a meta-holobiont. Under this framework, and to test the validity of the meta-holobiont concept, future studies will have to assess the degree of microbiota heritability and reciprocal exchange in reticulated clonal plants.

In the case of AM fungi, for example, a physical link produced by fungal hyphae and not the host plant can connect two plants (i.e., a common mycelium network [36]). However, in this case, the processes of microbial community filtering by the host are not in play, and integrated plastic responses at the meta-holobiont scale cannot be displayed. The mycorrhizal colonizer is in this case part of the immediate plant holobiont and it can also be part of an adjacent plant holobiont. A common mycorrhizal network thus induces particular interactions between two holobionts but not necessarily within a meta-holobiont. In addition, the selection pressures occurring on one plant in the network do not necessarily impact the other plants linked by a mycelial network.

Another case to consider is that of social animals organized in colonies. One example concerns social insects such as termites, which have developed active behaviors to promote microbiota exchanges. In this case, the social organization of the colonies serves as the support for microbiota, information, and resource exchanges. The applicability of the meta-holobiont concept in such an example will not be further developed here, but we encourage further discussions on this specific case where the strength and reliability of the social “conduit” is likely a key issue.

The inherent physiological integration of the meta-holobiont network and the transmission of microorganisms from mother to clonal offspring (35), and possibly reciprocally (i.e., from clonal offsprings to ascendant ramets), although not yet demonstrated, are clearly important to consider in the understanding of the clonal holobiont. If holobionts and hologenomes are units of biological organization for the observation and understanding of a given macroorganism (principles 1, 2, and 3 in reference 10), the meta-holobiont could be considered another level of organization for clonal macroorganisms.

## IMPACT OF MICROBIAL TRANSMISSION WITHIN THE META-HOLOBIONT

When resource distribution is patchy, clonal individuals may display plastic responses (Fig. 1). On the one hand, microbiota transfer within the clonal network may be an alternative to these plastic mechanisms, providing ramets with the ability to compensate for nutrient limitation (34). For instance, the transfer of AM fungi with high resource uptake ability at the ramet scale is probably less costly for the plants than developing an increased rooting system or increasing spacer length to forage for better patches. Specialization may then be seen in a wider context at the holobiont level (i.e., ramets that do specialize because of the recruitment of particular microbiota). Foraging through plant trait modifications would likely not be as efficient as capitalizing on microbiota functions (23, 34, 35). Note that such a trade-off between foraging plant traits and the use of microbial activity for resource uptake has already been described at the individual level regarding root development (e.g., see reference 36). Thus, our suggestion here at the meta-holobiont scale is only an extension of current considerations of individual plant foraging trade-offs.

On the other hand, foraging and specialization mechanisms may be indirectly mediated by the holobiont microbiota. Studies have demonstrated that microorganisms often manipulate plant traits (37), inducing changes in plant architecture (e.g., see reference 38) and biomass allocation (7, 39). In parallel, other studies demonstrated that a high diversity of AM fungi can reduce plant physiological integration in heterogeneous environments (39). These results suggest a reciprocity between plants and their microbiota in plastic adjustments to the environmental heterogeneity developed at the clonal fragment level.

In these two cases, the sharing of microorganisms between holobionts (i.e., ramets) within the meta-holobiont (i.e., network constituted of the set of all ramets) impacts both the holobiont and the meta-holobiont phenotypes (e.g., as explanation of the results in reference 34). The meta-holobiont concept may facilitate a better understanding of these integrated and plastic response mechanisms and more broadly of foraging strategies observed among clonal plants.

## HOLOBIONT DYNAMICS WITHIN THE META-HOLOBIONT NETWORK: APPLICATION OF METACOMMUNITY-BASED THEORIES

**FIG 3:**
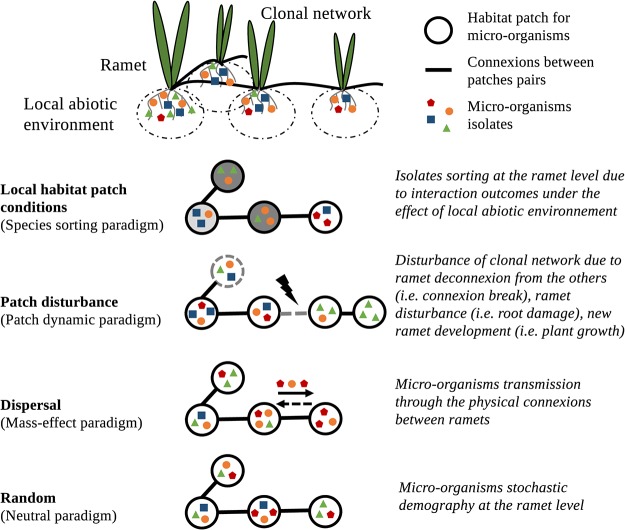
Links between meta-holobiont and meta-community. Clonal network comprises ramets connected by horizontal modified stems. Each ramet is under the influence of a local abiotic environment. Metacommunities are defined as a set of local communities linked by dispersal of multiple potentially interacting species (40). The figure presents the four main processes driving the assemblages at the metacommunity scale (local habitat patch conditions, patch disturbance, dispersal, and random processes) and the corresponding paradigms as defined by Leibold et al. (40). This is a simplified view of the metacommunity assembly rules, as all four processes interplay in each of the paradigms. Their transposition of the meta-holobiont scale is described on the right. Gray gradient indicates the habitat patch environmental conditions, and black arrows indicate the dispersal fluxes between a given habitat patch pair.

Microbiota transmission within the meta-holobiont has consequences at the holobiont scale. As previously discussed, the meta-holobiont could specialize if the environment is heterogeneous and thus alter the microbiota of individual holobionts. Conversely, a homogenization of holobionts’ microbiota within the meta-holobiont would be expected in homogeneous environments. This may condition the dynamics of microbiota assembly at the meta-holobiont level (Fig. 3). In the first case, holobionts represent heterogeneous pools of genomes that can be transferred within the meta-holobiont. In the second case, all holobionts represent the same potentialities. An understanding of microbiota assembly within meta-holobiont networks and its effect on the species dynamics of microorganisms therein is most likely to be achieved through consideration of metacommunity theories. A metacommunity is defined as a set of local communities linked by the dispersal of multiple species (40). Four different models of metacommunities have been described (see reference 40 for a review): patch-dynamic, species-sorting, mass effect, and neutral. These paradigms are distinguished by the processes governing species colonization and extinction in local communities within a metacommunity context (40). These models take into account the impacts of patch disturbances and of local biotic and abiotic conditions on species-sorting, the quantitative effects of dispersal on population size, and the community dynamics in stochastic metacommunity contexts. The transmission of microbiota within the meta-holobiont represents a flow of individuals affecting the interactions and demography of the local microbial communities (of a given holobiont in the meta-holobiont) (Fig. 3). Depending on the rates of microorganism dispersal between holobionts, and on the metacommunity model being considered, different effects would then be induced. When dispersal rates are low, primary effects with colonization events would be expected to regulate local community assembly (41). Conversely, if dispersal rates are high, mass and rescue effects would be expected (42), which would affect communities’ structures and dynamics (43). The application of metacommunity concepts (40) to holobiont theory should provide an interesting framework for understanding the impacts of meta-holobionts on microbial community assembly and dispersal in natural ecosystems.

## NETWORK THEORY AND META-HOLOBIONT PROPERTIES

From graph theory (i.e., the mathematical discipline that analyzes graph structure and its consequences), network analyses promote new understandings. For instance, the number of nodes, their connectance, and shape (i.e., modularity and nestedness) within a network affect the network’s resistance to perturbation and its resiliency, since these network characteristics determine individual fluxes at population and community levels (44). In plants in general, and particularly in clonal plants, the topology of modules has been shown to be shaped by the structural blueprint (i.e., basic structural organization or growth form), ontogeny, and plastic response to environmental conditions (45). Much research has been done on clonal plants to investigate how the network topology provides emergent functions to the clonal plant, such as the response to heterogeneous conditions, fluctuating environments, or disturbance (see reference 46 for a review). We frequently emphasize how host-microbiota interactions yield emergent properties. Here, these emergent properties are themselves intimately tied to the emergent functions of the clonal network. By transposing graph theories to the meta-holobiont concept, it may be possible to determine the properties of keystone holobionts or specific network structures that maximize the performance of the meta-holobiont. This could be achieved through the maximization of resilience based on the holobionts’ positions within the network or the degree of redundancy of microbiota compositions in the network. The meta-holobiont concept may thus provide a new way to consider these questions while taking into account the dynamic nature of multivarious plant-microorganism associations.

10.1128/mSystems.00213-18.1 TEXT S1 Different scales of clonality and definitions. Download Text S1, DOC file, 0.02 MB. Copyright © 2019 Vannier et al. 2019 Vannier et al. This content is distributed under the terms of the Creative Commons Attribution 4.0 International license.
