# Supplementary figures and images for: Clonal Plants as Meta-Holobionts
Source: mSystems. 2019 Mar 19;4(2):e00213-18. doi: 10.1128/mSystems.00213-18 (PMC6426648; doi:10.1128/mSystems.00213-18)

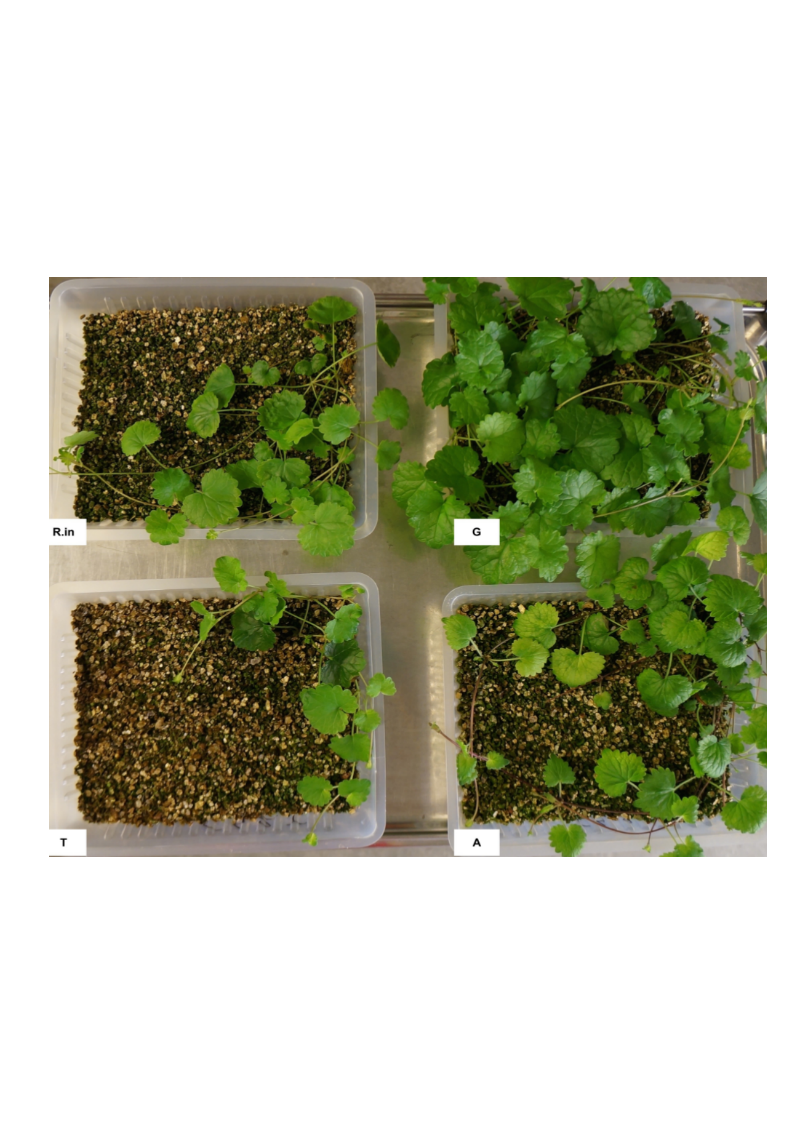

Supplement: FIG S1 [file mSystems.00213-18-sf001.tif]
