# Supplementary material for: Clonal Plants as Meta-Holobionts
Source: mSystems. 2019 Mar 19;4(2):e00213-18. doi: 10.1128/mSystems.00213-18 (PMC6426648; doi:10.1128/mSystems.00213-18)
Supplement: TEXT S1 [file mSystems.00213-18-s0001.doc]

**Supplementary Material :**

**The different scales of clonality, definitions**

*1st order module*: a non-independent growth unit of the plant linked to a particular function (leaf, root, bud, flower,…).

*2nd order module*: a set of 1st order module with a potential autonomy while separated from the other. 2nd order modules are also called ‘ramets’.

*Clonal fragment*: group of physically connected ramets. Ramets are connected through a horizontal modified stem (‘connexion’) that can grow aboveground (runner, stolon) or belowground (rhizome). Clonal fragments are also called a ‘clonal network’. Clonal fragments encompass a large range of network forms from aggregated clonal networks with high branching and low internode length (‘phalanx’ form) to loose networks with low branching and high internode length (‘guerilla’ form) (1; Fig. 2). Additionally, physical connexions between ramets have a certain lifespan (a few months (‘splitter’ strategy) to the whole plant life (‘integrator’ strategy) (2; Fig. 2). The physical network is then transitory for most plants and may cover space along a range of areas (centimeters to decameters).

*Integrated Physiological Unit*: group of physiologically connected ramets. Ramets are connected through a physical connexion but with an active transmission of molecules (hormones, signals, resources) through vessels. Information- and resource-sharing within the clonal network has been repeatedly demonstrated (3, 4). This physiological integration occurs for all first order modules within each second order module but also between all or part of the second order modules (5). Integration can last for a short period of time or for the life of the whole clonal fragment. A physical clonal fragment can correspond to potentially a unique physiological unit (‘integrated physiological unit’, 6) or to several of them as they become physiologically deconnected during clonal fragment growth.

*Clonal individual*: group of clonal fragments belonging to the same genet. A genet can extend over several hectares and last for thousands of years (7).

References for further informations

1. Lovett-Doust LL 1981. Population dynamics and local specialization in a clonal perennial (Ranunculus repens): I. The dynamics of ramets in contrasting habitats. J Ecol 69:743-755.

2. Oborny B, Czárán T, Kun A 2001. Exploration and exploitation of resource patches by clonal growth: a spatial model on the effect of transport between modules. Ecol Model 141:151-169.

3. Pitelka LF, Ashmun JW 1985. Physiology and integration of ramets in clonal plants. In: Population Biology and Evolution of Clonal Organisms, ed. Jackson JBC, Buss LW, Cook RE .Yale University Press, pp. 399–435.

4. Price EAC, Marshall C 1999. Clonal plants and environmental heterogeneity: an introduction to the proceedings. Plant Ecol 141:3–7.

5. Price EAC, Hutchings MJ 1992. The causes and developmental effects of integration and independence between different parts of Glechoma hederacea clones. Oikos 63: 376-386.

6. Watson MA, Casper BB 1984. Morphogenetic Constraints on pattern of carbon distribution in plants. Ann Rev Ecol Syst 15:233-258.

7. Harper JL 1977. Population biology of plants. Academic Press, London.
